# Plasma markers in pulmonary hypertension subgroups correlate with patient survival

**DOI:** 10.1186/s12931-021-01716-w

**Published:** 2021-05-04

**Authors:** T. Koudstaal, D. van Uden, J. A. C. van Hulst, P. Heukels, I. M. Bergen, L. W. Geenen, V. J. M. Baggen, A. E. van den Bosch, L. M. van den Toorn, P. P. Chandoesing, M. Kool, E. Boersma, R. W. Hendriks, K. A. Boomars

**Affiliations:** 1 Department of Pulmonary Medicine, Erasmus MC, University Medical Center, Doctor Molewaterplein 40, 3015 GD Rotterdam, The Netherlands; 2 Department of Cardiology, Erasmus MC, University Medical Center, Rotterdam, The Netherlands; 3 Department of Clinical Epidemiology, Erasmus MC, University Medical Center, Rotterdam, The Netherlands

**Keywords:** Pulmonary hypertension, Inflammation and immunity, Inflammatory cytokines, Survival and prognosis, Biomarkers, Pulmonary arterial hypertension, Chronic thromboembolic pulmonary hypertension

## Abstract

**Background:**

Recent studies have provided evidence for an important contribution of the immune system in the pathophysiology of pulmonary arterial hypertension (PAH) and chronic thromboembolic pulmonary hypertension (CTEPH). In this report, we investigated whether the inflammatory profile of pulmonary hypertension patients changes over time and correlates with patient WHO subgroups or survival.

**Methods:**

50 PAH patients (16 idiopathic (I)PAH, 24 Connective Tissue Disease (CTD)-PAH and 10 Congenital Heart Disease (CHD)-PAH), 37 CTEPH patients and 18 healthy controls (HCs) were included in the study. Plasma inflammatory markers at baseline and after 1-year follow-up were measured using ELISAs. Subsequently, correlations with hemodynamic parameters and survival were explored and data sets were subjected to unbiased multivariate analyses.

**Results:**

At diagnosis, we found that plasma levels of interleukin-6 (IL-6) and the chemokines (C-X3-C) motif legend CXCL9 and CXCL13 in CTD-PAH patients were significantly increased, compared with HCs. In idiopathic PAH patients the levels of tumor growth factor-β (TGFβ)*,* IL-10 and CXCL9 were elevated, compared with HCs. The increased CXCL9 and IL-8 concentrations in CETPH patients correlated significantly with decreased survival, suggesting that CXCL9 and IL-8 may be prognostic markers. After one year of treatment, IL-10, CXCL13 and TGFβ levels changed significantly in the PAH subgroups and CTEPH patients. Unbiased multivariate analysis revealed clustering of PH patients based on inflammatory mediators and clinical parameters, but did not separate the WHO subgroups. Importantly, these multivariate analyses separated patients with < 3 years and > 3 years survival, in particular when inflammatory mediators were combined with clinical parameters.

**Discussion:**

Our study revealed elevated plasma levels of inflammatory mediators in different PAH subgroups and CTEPH at baseline and at 1-year follow-up, whereby CXCL9 and IL-8 may prove to be prognostic markers for CTEPH patients. While this study is exploratory and hypothesis generating, our data indicate an important role for IL-8 and CXCL9 in CHD and CTEPH patients considering the increased plasma levels and the observed correlation with survival.

**Conclusion:**

In conclusion, our studies identified an inflammatory signature that clustered PH patients into WHO classification-independent subgroups that correlated with patient survival.

**Supplementary Information:**

The online version contains supplementary material available at 10.1186/s12931-021-01716-w.

## Introduction

Pulmonary hypertension (PH) is a debilitating disease characterized by structural remodeling of the arterial vasculature of the lung leading to increased vascular resistance and increased pulmonary arterial pressures, right ventricular (RV) hypertrophy, heart failure and ultimately, death [1]. In pulmonary arterial hypertension (PAH) patients, endothelial cell proliferation along with concurrent neoangiogenesis, when exuberant, results in the formation of glomeruloid structures in pulmonary arterioles known as the plexiform lesions [2–5]. PH is a heterogeneous disease, subdivided into five subgroups according to the WHO ERS/ESC classification [1]. Currently, PH-specific drugs are used to treat patients with PAH (WHO subgroup 1 PH) and inoperable chronic thromboembolic pulmonary hypertension (CTEPH) (WHO subgroup 4 PH), in contrast to WHO groups 2, 3 and 5, in which only the underlying diseases can be treated [1]. However, even with PH-specific treatment strategies, survival remains poor with a mean 5-year survival of ~ 60% for PAH [6, 7] and CTEPH patients [7–9].

Over the years, accumulating evidence points to a pathological role for the immune system in PAH and CTEPH [10–12]. Lungs of idiopathic PAH (IPAH) patients (belonging to WHO subgroup 1 PH) display an increased inflammatory mark consisting of T and B lymphocytes, mast cells, dendritic cells and macrophages [13, 14]. Furthermore, activation of B lymphocytes and circulating auto-antibodies were found in PAH patients [15–17]. Thrombotic lesions in CTEPH patients contain activated T and B lymphocytes, macrophages and neutrophils and patients display elevated levels of circulating cytokines and chemokines [18, 19]. These inflammatory mediators can contribute directly to recruitment of immune cells, activation and proliferation of pulmonary arterial smooth muscle cells, and endothelial dysfunction. A very recent unbiased whole-blood transcriptome analysis in PAH patients and healthy controls (HCs) [20] identified a signature of 507 PAH-associated genes, in which T cell signaling, phosphoinositide 3-kinase (PI3K) signaling in B lymphocytes and interleukin-6 (IL-6) signaling were among the top canonical pathways.

In cross-sectional studies of PAH patients, increased IL-6, IL-8 and IL-10 in serum correlated with reduced survival and quality of life [21, 22]. Increased levels of circulating pro-inflammatory cytokines were also found in CHD-PAH and CTD-PAH [23–25]. In an unsupervised analysis of blood cytokine profiles of PAH patients, different immune phenotypes were distinguished with different clinical risk profiles, independent of WHO PH subgroups [26]. Accumulating evidence supports a major role for IL-6, considering that IL-6 receptor (IL-6R) expression and signalling is crucial for PAH development and progression [27] and that circulating IL-6 associates with specific clinical phenotypes and outcomes in various PAH subgroups [28]. Increased transforming growth factor (TGF)β receptor signalling and decreased Bone morphogenetic protein receptor type II (BMPR2) signalling were shown to contribute to PAH pathogenesis [29].

Serum of PAH patients contains increased levels of the CXCL9 chemokine, which is involved in the differentiation of IFNγ-producing T-helper 1 (Th1) cells expressing its receptor CXCR3 [26, 30]. Likewise, in PAH and CTEPH serum samples and lung tissue an increase was found of CXCL13 [31], which is implicated in the organization of B cells in follicles and germinal centers because its receptor is expressed on B cells and follicular T-helper cells. Levels of vascular endothelial growth factor (VEGF) are increased in PAH patients during treatment and are associated with risk of death and hospitalization at 16-week follow-up [32].

Nevertheless, many questions remain unanswered. Currently, limited data are available on the levels of cytokines or chemokines in treatment-naïve patients, particularly in CTEPH, on changes in cytokine and chemokine levels during follow-up and on the possible correlation of inflammatory marker signatures with prognosis. Therefore, our aim was to study circulating inflammatory markers in PAH and CTEPH patients at diagnosis and at 1-year follow up. To the best of our knowledge, our study is the first to investigate different subgroups of PAH and CTEPH patients together. We performed unsupervised clustering of inflammatory profiles and correlated these to transplant-free survival.

## Materials and methods

### Patients and study design

This prospective observational cohort study was conducted between May 2012 and July 2019. PH patients > 18 years old with a mean pulmonary arterial pressure (mPAP) ≥ 25 mmHg, a wedge pressure ≤ 15 mmHg and a PVR ≥ 3WU measured by right heart catheterization were invited to take part in the study at diagnosis and a large majority agreed [33]. PAH and CTEPH patients were diagnosed according to the ERS/ECSC guidelines [1]. Patients were subdivided according to the World Health Organization (WHO) classification in 16 idiopathic PAH (IPAH), 24 connective tissue disease-associated PH (CTD-PAH), 10 congenital heart disease-associated PH (CHD-PAH)) and 37 CTEPH (Table 1) [1, 33].

**Table 1:** Demographic and patient characteristics

	IPAH (n = 16)	CTD-PAH (n = 24)	CHD-PAH (n = 10)	CTEPH (n = 37)	HC (n = 18)	*p* value
Baseline clinical characteristics						
Gender, female (%)	12 (75%)	21 (88%)	4 (40%)	20 (54%)	9 (50%)	
Age, y	54.3 ± 17.2	63.6 ± 11.8	41.0 ± 17.7	61.4 ± 14.2	31.6 ± 9.9	< 0.0001
BMI, kg/m^2^	27.7 ± 8.0	27.1 ± 4.4	23.7 ± 4.8	28.8 ± 6.0		0.16
NYHA class 3–4, n (%)	12 (75%)	15 (63%)	3 (30%)	17 (46%)		
6MWT, m	350 ± 135	333 ± 122	426 ± 173	379 ± 129		0.35
NT-pro BNP, pmol/L	317 ± 467	519 ± 1037	65 ± 88	127 ± 199		0.07
Underlying CTD						
SSc, n (%)		20/24 (83%)				
SLE, n (%)		4/24 (17%)				
Baseline right heart catheterization						
mPAP, mmHg	58.9 ± 16.5	41.5 ± 12.5	43.11 ± 14.9	40.1 ± 12.6		0.0001
mRAP, mmHg	11.9 ± 6.7	10.4 ± 6.0	10.8 ± 6.2	9.6 ± 7.1		0.72
Capillary wedge pressure, mmHg	9.2 ± 4.2	12.9 ± 8.1	14.5 ± 6.6	12.3 ± 4.5		0.21
PVR, wood units	10.6 ± 3.9	6.0 ± 3.5	4.3 ± 2.9	5.3 ± 3.4		0.0002
PH-Medication						
At baseline, n (%)	0/16 (0%)	0/24 (0%)	0/10 (0%)	0/37 (0%)		
At 1 year follow up						
No PH-medication	0/13 (0%)	0/11 (0%)	2/6 (33%)^3^	3/19 (16%)^5^		
Mono therapy, n (%)	1/13 (8%)^1^	1/11 (9%)^2^	1/6 (17%)^4^	11/19 (58%)		
Duo therapy, n (%)	6/13 (46%)	9/11 (82%)	3/6 (50%)	5/19 (26%)		
Triple therapy, n (%)	6/13 (46%)	1/11 (9%)	0/6 (0%)	0/19 (0%)		
Immunomodulatory drugs						
At baseline, n (%)	0/16 (0%)	3/24 (13%)	0/10 (0%)	0/37 (0%)		
At 1 year follow up, n (%)	0/13 (0%)	3/11 (27%)	0/6 (0%)	0/19 (0%)		
Survival						
Death/lung transplant < 3 years	2 (12.5%)	8 (33.3%)	0 (0%)	6 (16.2%)		
Death/lung transplant > 3 years	2 (12.5%)	0 (0%)	3 (30%)	2 (5.4%)		

Data given as ‘mean, ± SD’, unless otherwise indicated

*BMI* body mass index, *CTEPH* chronic thromboembolic pulmonary hypertension, *PAH* pulmonary arterial hypertension, *IPAH* idiopathic pulmonary arterial hypertension, *CHD* congenital heart disease, *CTD* connective tissue disease, *6MWT* 6-min walk test, *NT-pro BNP* The N-terminal prohormone of brain natriuretic peptide, *SSc* systemic sclerosis, , *SLE* systemic lupus erythematosus, *mPAP* mean pulmonary arterial pressure, *mRAP* mean right atrium pressure, *PVR* pulmonary vascular resistance, *RV* right ventricle, *RA* right atrium, *RVSP* right ventricular systolic pressure

^1^This IPAH patient was on ERA monotherapy, due to severe side-effects on PDE5 therapy

^2^This CTD-PAH patient was on PDE5 monotherapy due to severe side-effects on ERA therapy

^3^These CHD-PAH patients were not started on PAH-medication immediately, since in one patient a possible effect of a surgical correction was awaited, and the other patient was started on medication after one-year since she had persistent PAH

^4^This CHD-PAH patient was on PDE5 monotherapy since the PAH was mild

^5^These CTEPH patients were not started on PH-medication since they underwent a pulmonary endarterectomy

Similar to prior work from our group [34], exclusion criteria were incomplete diagnostic work-up and therefore no confirmed PH diagnosis, not treatment-naïve, age < 18 years, or not capable of understanding or signing informed consent. The study protocol was approved by the medical ethical committee. A written informed consent was provided by all patients. This study was performed conform the principles outlined in the Declaration of Helsinki.

### Clinical data collection

Clinical data were collected during the inpatient screening visit for analysis of PH [34]. All patients underwent physical examination by a cardiologist and a pulmonary physician, 6-min walking test, spirometry, VQ scan, chest computed tomography scan, 12-lead electrocardiography (ECG), echocardiography, venous blood sampling and right heart catheterization. Patient characteristics and vital signs were collected, including age, sex, height, weight, systemic blood pressure, heart rate and peripheral oxygen saturation. The New York Heart Association (NYHA) functional class was used to grade the severity of functional limitations by the presence of signs and symptoms of heart failure. During right heart catheterization, a Swan-Ganz catheter was inserted in the internal jugular vein. A standardized protocol for the work-up of PH was used to obtain hemodynamic measurements and thermodilution or Fick’s principle was used to measure cardiac output [1]. If the obtained capillary wedge pressure was ambiguous, a fluid challenge was performed to distinguish pre-capillary PH from PH due to left heart disease. Data were collected and stored in PAHTool (version 4.3.5947.29411, Inovoltus, Santa Maria da Feira, Portugal), an online electronic case report form.

### Clinical follow-up and definition of endpoints

Patients were treated according to the ERS/ESC guidelines [1] and prospectively followed-up by half-yearly scheduled visits to the outpatient clinic. CTEPH patients were assessed for eligibility for either a pulmonary endarterectomy or a balloon pulmonary angioplasty. In our CTEPH cohort, 7 patients underwent a pulmonary endarterectomy treatment in the period following after the baseline blood sampling. Patients who underwent one of the above procedures were not censored afterwards. The primary composite endpoint was defined as all-cause mortality or lung transplantation. Patients were continuously included in our study, with a mean follow-up duration of 39.5 months.

### Inflammatory cytokine and chemokine assessment

At baseline and at every half-yearly follow-up visit (up to 10-year follow-up), peripheral venous blood samples were collected and processed within 2 h by Ficoll separation and divided into plasma and peripheral blood mononuclear cells (PBMCs) fractions. Plasma samples were subsequently stored at -80 degrees.

The concentrations of inflammatory markers (VEGF-A, TGF*β*, CXCL-9, CXCL-13, IL-1*β*, IL-6, IL-8, IL-10) in plasma were determined in duplicate by ELISA (R&D systems Europe, Abingdon, UK) (Additional file 1: Table 1). Streptavidin-HRP (eBioscience) and tetramethylbenzidine (TMB) substrates (eBioscience) were used to develop the ELISA. Optical densities were measured at 450 nm using a Microplate Reader (Bio-Rad, Hercules, CA, USA).

### Statistical analysis, principal component analysis and multiple factor analysis

Statistical evaluation of baseline cytokine and chemokine measurements in IPAH, CTD-PAH, CHD-PAH and CTEPH patients was performed using a Kruskal Wallis test. Next, we compared multiple groups using Dunn’s multiple comparison test, leading to separate p values for each comparison between two subgroups. Paired baseline and 1-year follow-up cytokine data were analyzed using a Wilcoxon signed rank test. The Kaplan–Meier method was applied to estimate the cumulative primary endpoint-free survival (based on all-cause mortality or lung transplantation) function. All statistical tests were two-sided and p-values < 0.05 were considered statistically significant. Statistical analyses were performed using Prism (GraphPad Software, La Jolla, CA, USA) or SPSS version 24.

Principal component analysis (PCA) and multiple factor analysis (MFA) were performed using R and RStudio, and the packages FactoMineR and Factoextra [35]. Missing data were imputed using the R package MissMDA [36]. Missing variables were imputed using the R package. Only imputed variables with a small area of variability and thereby high credibility in the multiple imputation method were used for analysis. Prior to PCA and MFA analysis ELISA data were log10 transformed to better fit a normal distribution and were scaled. Contribution of the variables to the PCs was determined in percentages by (squared cosine of the variable*100)/(total squared cosine of the principal component). The number of dimensions to be interpreted were determined by the R package FactoInvestigate. Dimensions with an inertia higher than the inertia obtained by a random distribution, therefore providing the best representation of the data variability, were considered. Patients were labelled by ≥ 3-year survival or deceased/transplanted < 3 years after diagnosis to determine clustering of individuals on the first and second PCs.

The variability explained by the PCA was tested for statistical significance by inertia of the first two dimensions using the R package FactoInvestigate. Separation of HC, PAH and CTEPH patients was tested using a 1way ANOVA test with a Kruskal Wallis test combined with a Dunn’s multiple comparison test of Dim1 and/or Dim2 coordinates in Prism. Statistical evaluation of separation between ≥ 3-year survival or deceased/transplanted < 3 years after diagnosis in PCAs and MFAs was tested using a Mann–Whitney U test of Dim1 coordinates of alive versus deceased/transplanted individuals in Prism.

## Results

### Analysis of inflammatory mediators in treatment-naïve PAH and CTEPH patients at diagnosis

**Fig. 1:**
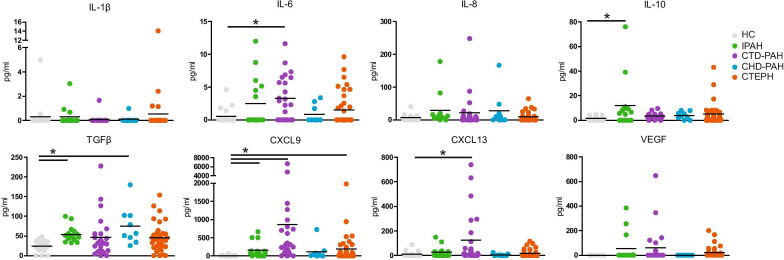
Inflammatory markers in plasma of treatment-naïve PAH and CTEPH patients at diagnosis. The indicated inflammatory markers were measured in plasma samples from 50 PAH patients (16 IPAH, 24 CTD-PAH and 10 CHD-PAH), 37 CTEPH patients at diagnosis and 18 healthy controls (HC) by ELISA. Data are shown as symbols for individual patients or HCs; horizontal bars represent mean values. Statistical analysis was performed using a Kruskal–Wallis test combined with a Dunn’s multiple comparison test. * = p < 0.05

Fifty PAH patients (16 IPAH, 24 CTD-PAH and 10 CHD-PAH), 37 CTEPH patients and 18 healthy controls (HC) were included (Table 1). Plasma from patients at diagnosis and HCs were analyzed for the cytokines IL-1β, IL-6, IL-8, IL-10 and TGFβ, the chemokines CXCL9 and CXCL13 and VEGF (Fig. 1). Compared with HCs, plasma levels of IL-6 and IL-10 were significantly elevated in CTD-PAH and IPAH patients, respectively. TGFβ was significantly increased in both IPAH and CHD-PAH patients. The CXCL9 chemokine was elevated in IPAH, CTD-PAH and CTEPH patients, whereas CXCL-13 was only increased in CTD-PAH patients, when compared with HCs. No significant differences between HCs and any of the four patient groups were observed for IL-1β, IL-8 and VEGF.

In summary, at diagnosis we found significantly increased plasma levels of IL-10, TGFβ*,* and CXCL9 in IPAH patients, of IL-6, CXCL9 and CXCL13 in CTD-PAH patients, and of CXCL9 in CTEPH patients.

### Correlation of inflammatory mediators at diagnosis with hemodynamic parameters and survival

We did not find significant relations between plasma levels of inflammatory mediators at baseline and hemodynamic parameters including pulmonary arterial pressure (mPAP), mean right atrial pressure (mRAP), pulmonary vascular resistance (PVR) and N-terminal pro B-type natriuretic peptide (NT-pro BNP), consistent with reported findings in a cross-sectional study [21] (data not shown).

**Fig. 2:**
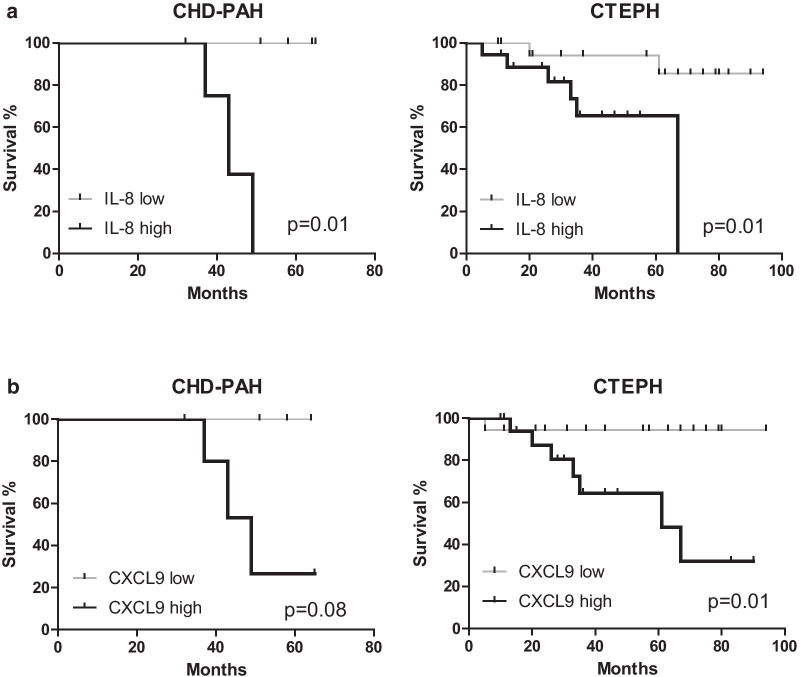
Effects of IL-8 and CXCL9 plasma levels on patient survival. Kaplan-Meyer survival analyses for **a** IL-8-high/low subgroups and **b** CXCL9-high/low of the indicated WHO PH patient subgroups. Statistical analysis was performed using a log-rank (Mantel-Cox) test and a Gehan-Breslow-Wilcoxon test. p Values are shown for the (Mantel-Cox) test

Next, we explored potential correlations between inflammatory markers and patient survival by Kaplan–Meier analyses, whereby for each patient group two subgroups were defined with above or below median values for the inflammatory marker. At the time of censoring (mean follow-up duration of 39.5 months), 22 out of 87 patients had died without undergoing lung transplantation and two patients had received a lung transplantation. When we analyzed survival (all-cause mortality and/or lung transplantation), significant differences were found for IL-8 and CXCL9. CHD-PAH and CTEPH patients with high levels of IL-8 at baseline showed a significantly reduced survival compared with IL-8^low^ patients (p = 0.013 and p = 0.016, respectively; Fig. 2a). Similar results were obtained when we compared CXCL9^high^ and CXCL9^low^ patients, whereby significance was reached for CTEPH but not for CHD-PAH patients (p = 0.011 and 0.083, respectively) (Fig. 2b). In IPAH and CTD-PAH patients, no significant differences were found (Additional file 1: Fig. 1). A similar analysis for time to clinical worsening (TTCW), defined as > 15% decline in 6mwt, admission to the hospital for PH related complications, or the need for increase of PH specific medication or the start of increase of diuretics) only revealed a significant difference for CXCL9 in CTEPH patients (data not shown).

### Comparison of inflammatory mediators at diagnosis and at 1-year follow-up

**Fig. 3:**
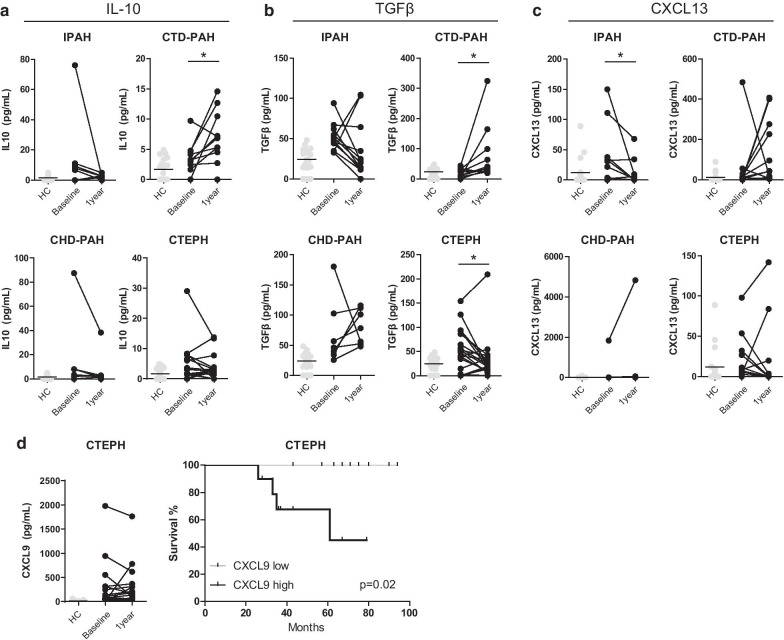
Inflammatory markers in plasma of PAH and CTEPH patients at diagnosis and 1-year follow up. **a**–**c** Paired plasma concentrations for interleukin IL-10 (**a**), TGFβ (**b**) and CXCL13 (**c**) measured by ELISA, for a subset of patients from the indicated WHO PH subgroups, at diagnosis and at 1-year follow-up, compared with HCs. Data are shown as symbols for individual patients or HCs. **d** Paired plasma cytokine measurements by ELISA for CXCL9 at diagnosis and at 1-year follow-up for CTEPH patients and Kaplan Meyer survival analyses, starting at 12 months follow up, for CXCL9-high/low subgroups of CTEPH patients at 1-year follow up. Data are shown as symbols for individual patients or HCs; horizontal bars represent mean values; connecting lines between baseline and 1 year follow up samples indicate paired same-patient samples. Statistical analysis was performed using a Wilcoxon signed-rank test for the ELISA measurements and for the survival analysis a log-rank (Mantel-Cox) test and a Gehan-Breslow-Wilcoxon test was performed. * = p < 0.05

To follow circulating inflammatory mediators over time, plasma levels were measured at 1-year follow-up in 31 PAH patients (13 IPAH, 11 CTD-PAH and 6 CHD-PAH) and 19 CTEPH patients and compared to their baseline values. For IL-1β, IL-6, IL-8, CXCL-9 and VEGF, no significant changes were found for any of the four patient groups (shown for IL-1β and IL-6 in Additional file 1: Fig. 2). Strikingly, a significant increase in IL-10 and TGFβ at 1-year follow-up compared to baseline was observed in CTD-PAH patients (Fig. 3a, b). CTEPH patients showed a significant decrease in TGFβ levels. CXCL-13 was significantly decreased after 1-year follow-up in IPAH patients only (Fig. 3c).

Only CXCL9 after 1-year follow up showed a significant correlation with survival in CTEPH patients (Fig. 3d). For the other inflammatory markers or the delta values (difference between 1-year follow-up and baseline values) no significant changes were observed (data not shown).

Taken together, these results show that although in PH patients plasma levels of IL-1β, IL-6, IL-8, CXCL-9 and VEGF were dynamic, we did not observe a significant increase or decrease in any of the patient groups. By contrast IL-10, CXCL13 and TGFβ showed disease group-specific changes at 1-year follow-up compared with baseline.

### Principal component analyses of inflammatory markers in WHO PH subgroups

**Fig. 4:**
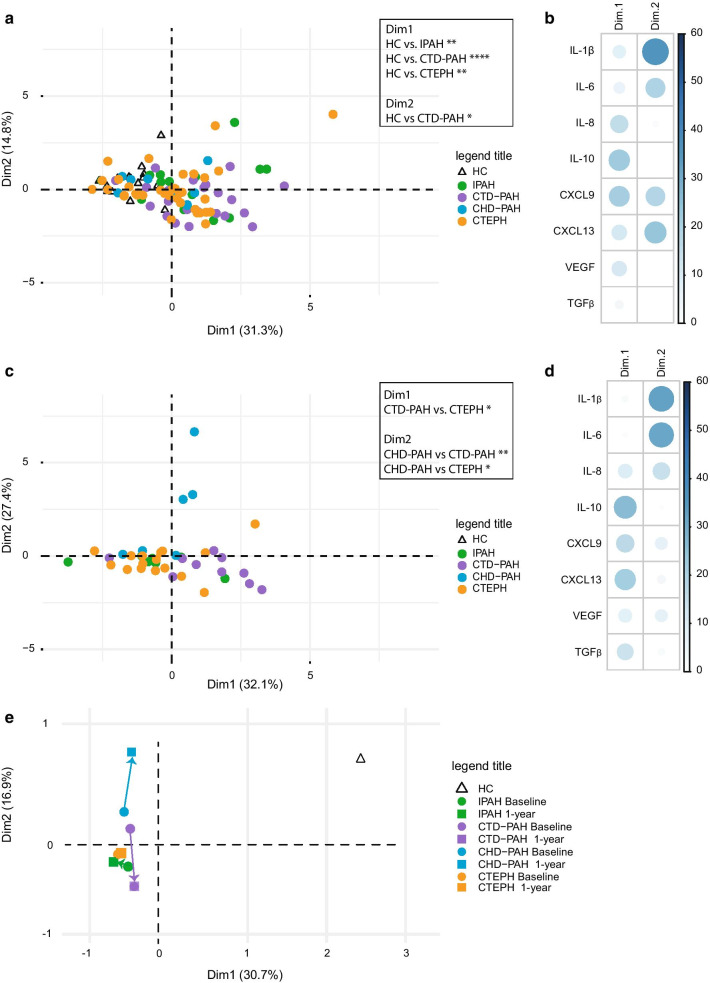
Principle component analysis of inflammatory markers in plasma of PAH and CTEPH patients. **a**–**d** Unsupervised principle component analysis (PCA) of inflammatory markers, measured by ELISA, in plasma of healthy controls (HC) and the indicated WHO PH patient subgroups at diagnosis (**a**, **b**) and at 1-year follow-up (**c**, **d**). PCAs were on log10-transformed and scaled concentrations values; each symbol point represents an individual patient or HC sample (**a**, **c**). Representation of the contribution in percentages of the variables on the first (Dim.1) and second (Dim.2) principal component at diagnosis (**b**) and at 1-year follow-up (**d**). The blue color range indicates the contribution to the principal components. **e** PCA plot of average coordinates of Dim1 and Dim2 for the indicated WHO PH patient subgroups. Arrows between the dots represent the PC change from diagnosis to 1-year follow up. Statistical analysis was performed by a one-way ANOVA (Kruskal–Wallis test) combined with a Dunn’s multiple comparison test. * = p < 0.05, ** = p < 0.01

To obtain a more comprehensive overview of the inflammatory marker profiles across the four patient groups and HCs, we performed principal component analyses (PCA), which reduced the dimensionality of the data set. The PCA of the eight inflammatory markers showed a non-random distribution over Dim1 and Dim2, which was not due to gender or age, and did not separate the WHO PH subgroups classified on the basis of etiology and predisposing factors [1] (Fig. 4a and data not shown). For each WHO PH subgroup the individual patients were quite scattered over the PCA plot, whereby Dim1 revealed a modest but significant separation of HCs from IPAH, CTD-PAH and CTEPH patients (Fig. 4a). No significance difference was found between HCs and CHD-PAH patients, consistent with our finding that CHD-PAH patients had a plasma inflammatory profile that was similar to that of HCs (Fig. 1) except for TGFβ, which did not substantially contribute to Dim1 or Dim2 (Fig. 4b). Whereas the impact of IL-8, IL-10 and CXCL9 was dominant in Dim1, IL-1β, IL-6 and the two chemokines dominated Dim2 (Fig. 4b).

Furthermore, we performed a PCA on 1-year follow up cytokine levels. The inflammatory profile of CTD-PAH patients was separated from CTEPH patients by Dim1, to which particularly IL-10, CXCL9 and CXCL13 levels contributed (Fig. 4c, d). Dim2 separated CHD-PAH patients from CTD-PAH and CTEPH, whereby IL-1β and IL-6 showed a major contribution.

To compare baseline and 1-year follow up samples for each WHO PH subgroup, we performed a PCA that included HCs and those patients for which baseline and 1-year follow up measurements were available (Additional file 1: Fig. 3A, B). This PCA revealed that HCs were clearly separated from all PH subgroups, which clustered together. Subsequently, we determined and plotted the average Dim1 and Dim2 coordinates of the HCs and the two time points for each WHO PH subgroup (Fig. 4e). This analysis revealed that for IPAH and CTEPH the differences between baseline and 1-year follow-up were limited. In contrast, CTD-PAH and CHD-PAH patients showed clear changes over time for Dim2, to which IL-1β contributed most (Additional file 1: Fig. 3A), but in opposite directions. We did not find evidence for a normalization of the inflammatory markers towards the HC profile.

### Inflammatory profile and clinical parameters correlate with PH patient survival

**Fig. 5:**
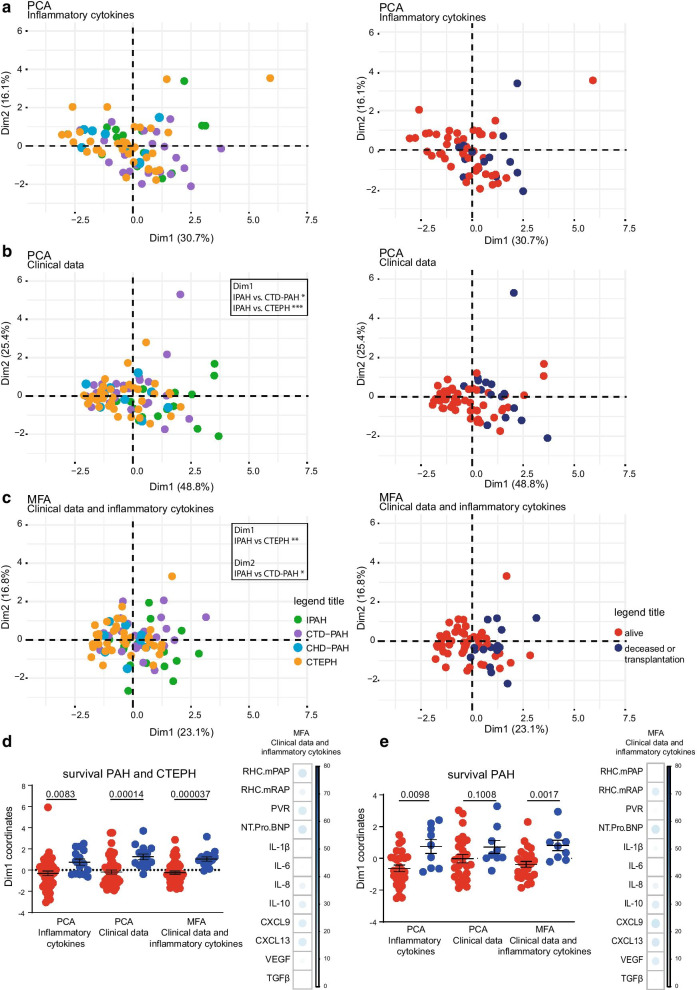
PAH and CTEPH patients cluster based on survival in multivariate analyses. **a**, **b** Unsupervised principal component analysis (PCA) of inflammatory markers measured by ELISA in plasma (**a**) and of clinical parameters (**b**), showing the indicated WHO PH patient subgroups (*left*) or subgroups of survival of > 3 years (alive) or < 3 years (deceased/transplantation) (*right*). **c** Multiple factor analysis (MFA) combining clinical data and log10 transformed and scaled plasma inflammatory marker concentrations, showing the indicated patient subgroups (*left*) or survival of > 3 years (alive) or < 3 years (deceased/transplantation) (*right*). **d**, **e**. Dim1 coordinate values showing the separation between survival of > 3 years (alive) or < 3 years (deceased/transplantation) for the indicated PCAs and MFAs of PAH and CTEPH patients (**d**) or PAH patients alone (**e**) and contribution of the variables for Dim1 to the MFA. Statistical analysis between PH groups was performed by a one-way ANOVA (Kruskal–Wallis test) combined with a Dunn’s multiple comparison test. * = p < 0.05, ** = p < 0.01, *** = p < 0.001. Separation between survival groups was evaluated using a Mann–Whitney U test on principal component 1 coordinates of alive versus deceased/transplantation. p Values are indicated

We aimed to explore whether the inflammatory profiles would correlate with survival, independent of WHO subgroup classification. We first performed a PCA without including HCs. The obtained pattern was quite similar to the one that did include HCs, both regarding the weak separation between CHD-PAH and the other three PH subgroups (Fig. 5a; compare with Fig. 4a) and the inflammatory mediators that contributed most to Dim1 and Dim2 (Additional file 1: Fig. 4A; compare with Fig. 4b). To link inflammatory profiles to survival, patients were divided into two subgroups defined by > 3 years survival after diagnosis or < 3 years survival or lung transplant within 3 years after diagnosis. These two survival subgroups were significantly separated in the PCA based on Dim1 (p = 0.0083; Fig. 5a, d) in which the impact of IL-8, IL-10 and CXCL9 was dominant (Additional file 1: Fig. 4A).

In parallel, a PCA was performed using four clinical parameters including mPAP, mRAP, PVR and NT-proBNP, resulting in significant separation of IPAH versus CTD-PAH and CTEPH, and a significant clustering of survival subgroups in Dim1, which was dominated by mPAP and PVR (p = 0.00014; Fig. 5b, d; Additional file 1: Fig. 4B). Finally, a multiple factor analysis (MFA) was performed using both clinical and inflammatory parameters, which resulted in a significant separation of IPAH and CTEPH patients in Dim1 and of IPAH and CTD-PAH patients in Dim2 (Fig. 5c). Importantly, the combination of parameters yielded the best separation of the two survival groups in Dim1 (p = 0.000037) with a large impact of mPAP, PVR, CXCL9 and CXCL13 (Fig. 5c, d).

Since CTEPH patients have a better prognosis than PAH patients [7–9], we additionally performed the PCA and MFA analyses with PAH patients only. Again, the two survival groups were separated in Dim1, which reached significance in the PCA for inflammatory markers (p = 0.0098) and in the MFA for the combination of clinical and inflammatory markers (p = 0.017) (Additional file 1: Fig. 5A; Fig. 5E). Hereby, the dominant mediators in Dim1 were similar to those in the analyses above that did include CTEPH patients (Additional file 1: Fig. 4C). In the combined MFA CXCL9, mRAP and NT pro BNP had the largest contribution to Dim1 (Fig. 5E).

In the PCA and MFA analyses of either PAH and CTEPH patients combined or in PAH patients alone, Dim2 was not able to significantly separate survival groups (Additional file 1: Fig. 5D, E).

Taken together, these findings show that multivariate data analysis using a combination of inflammatory markers and clinical parameters most robustly clustered PH patients into WHO classification-independent subgroups that significantly correlated with patient survival.

## Discussion

We investigated inflammatory markers at different time-points in PAH and CTEPH patients, performed unsupervised clustering by PCA and correlated inflammatory profiles to transplant-free survival. We found significantly increased plasma levels of IL-10, TGFβ*,* and CXCL9 in IPAH patients, of IL-6, CXCL9 and CXCL13 in CTD-PAH patients, and of CXCL9 in CTEPH patients at diagnosis. Our analyses revealed lower levels of several circulating cytokines in our IPAH patients at diagnosis compared to previous reports of cross-sectional data [21, 37]. Possibly, this is indicative for existing heterogeneity between patients, different pathophysiological changes over time during disease progression or therapeutic effects due to PAH-specific therapy.

In CTD-PAH patients IL-10 and TGFβ levels increased significantly compared to baseline levels after one year of therapy. Likewise, CXCL13 levels in IPAH patients decreased significantly compared to baseline levels. Our multivariate analyses suggested that the inflammatory profile changes over time: in CHD-PAH patients Dim2 shifted in the direction of the HC, whereas in CTD-PAH patients Dim2 shifted away from HCs. This may be linked to therapy or due to the natural course of pathophysiology in these patients. In this context, it is of note that there is growing evidence for anti-inflammatory and anti-aggregation activity of the phosphodiesterase type 5 inhibitor sildenafil [38]. Nevertheless, we did not find correlations between changes in cytokine or chemokine levels over time and patient survival, indicating that these changes are most probably not prognostic for disease outcome.

In CETPH patients, high levels of CXCL9 and IL-8 at baseline correlated with decreased survival. CXCL9 is a known regulator of immune cell migration, differentiation and activation and is required for optimal Th1 cell differentiation and IFNγ production by T cells in vivo [30]. The receptor for CXCL9 is CXCR3, which is a marker for Th1 cells and IFNγ-producing Th17 cells, also known as Th17.1 cells [39]. Previous research has shown that PAH patients display Th17 cell immune polarization [40]. Possibly, by endovascular triggers in CTEPH, CXCL9 is upregulated for the recruitment of cytotoxic lymphocytes, natural killer cells and macrophages. Moreover, CXCL9 is known to be involved in activation of immune cells in response to IFNγ. CXCL9 may prove to be a biomarker reflecting pathological involvement of the immune system in CTEPH patients.

Similar to CXCL9, also IL-8, a known chemokine produced by macrophages and other cell types such as epithelial cells, showed a negative correlation with survival in CTEPH patients. In contrast to CXCL9, which is a natural inhibitor of angiogenesis, IL-8 is a pro-angiogenic factor also known as a chemoattractant for immune cells to the site of endovascular damage. Previous studies have shown increased levels of IL-8 in CTEPH patients on treatment [19, 41, 42]. To the best of our knowledge, our study is the first to show that—although high levels of IL-8 at baseline correlate with decreased survival in these patients—IL-8 was not increased in all CTEPH patients analyzed at baseline.

In accordance to previous studies, we found increased levels of IL-6 in a majority of CTD-PAH patients at baseline, as well as in a subgroup of IPAH patients. In contrast to earlier cross-sectional studies in IPAH patients [21], we did not find a correlation of IL-6 with survival in any of the PAH subgroups or CTEPH patients. This might be indicative for the pathological role of IL-6 during disease progression, it might however also be a secondary or a bystander effect. In our data, we did not find a correlation between changes over time for IL-6 and survival. Furthermore, IL-6 did not display a major role in the distinction between < 3 and > 3-year survival of PAH patients in our multivariate analyses. Currently, a clinical trial with anti-IL6 treatment in PAH patients is ongoing to further elaborate the possible pathological role for IL-6 [43].

Except for TGFβ, our cohort of CHD-patients displayed no significant increases in circulating cytokines. In apparent contrast, a previous study identified a minor increase of endothelin-1, IL-1β, IL-6, IL-8, tumor necrosis factor α and VEGF, but a significant correlation with lung function was not observed [23]. Our PCA or MFA did not separate CHD-PAH patients and HCs, supporting the notion that inflammation does not play a significant role in the pathogenesis of PH in CHD-patients.

However, in the PCA a clear distinction between HCs and PAH or CTEPH patients was observed, together with an immunological overlap between the different PAH subgroups. A subgroup of IPAH patients shared immunological features with CTD-PAH patients. Considering that in ~ 40% of IPAH patients specific vascular autoantibodies were found [15–17], it is conceivable that this IPAH subgroup has a more autoimmune phenotype. Our MFA of inflammatory markers and clinical data revealed significant differences between IPAH and CTEPH (dim 1) and between IPAH and CTD-PAH (dim 2), but not between IPAH and CHD-PAH, indicating differential involvement of the immune system in disease pathology of PAH subgroups.

A key finding in our multivariate analyses was that combined profiling using both inflammatory and clinical parameters provided the most significant distinction for patient survival. We could exclude the relatively good prognosis for CTEPH as a dominant factor in this survival analysis, because our sub-analysis that included only PAH patients showed a comparable significant distinction of the < 3 and > 3-year survival groups. Interestingly, in this sub-analysis of the three PAH patient groups, clinical data alone did not provide a significant distinction in survival. Rather, levels of cytokines and particularly of the chemokines CXCL9 and CXCL13 appeared to be major determining factors in survival. Previously, it has been shown that CXCL9 and several CC-family chemokines important for chemotaxis of myeloid cells play a central role in distinguishing clusters of PAH immune phenotypes with different clinical risks [26] The finding that CXCL13 is one of the markers that could be linked to survival in our PCA/MFA analysis (Fig. 5d) may support a critical role of B cell recruitment and organization in follicles and germinal centers in PAH. This would be consistent with the identification of bronchus-associated lymphoid structures [14] as well as circulating auto-antibodies in PAH patients [15–17]. Nevertheless, it has been reported that on its own serum CXCL13 only showed a weak association with markers of disease severity [31].

There are some limitations to our study. Firstly, while this study is exploratory and hypothesis generating, our data indicate an important role for IL-8 and CXCL9 in CHD-PAH and CTEPH patients considering the increased plasma levels and the observed correlation with survival. Due to the prospective design of our study, not all patients reached a follow-up duration of > 3 years; this may have led to limited survival events. Furthermore, a survival bias may have occurred, because only patients who survived for > 1 year were included in our paired 1-year follow-up measurements. Lastly, while our CTEPH cohort consisted of 37 patients, our PAH cohort was rather limited in size when stratified into the different PAH subgroups. Nevertheless, for many inflammatory markers we were able to detect significant differences between individual PAH subgroups and healthy controls.

In summary, we found significantly increased plasma levels of various cytokines in three PAH subgroups and CTEPH patients. Particularly when inflammatory mediators were combined with clinical parameters, PCA and MFA multivariate analyses clustered PAH and CTEPH patients into WHO classification-independent subgroups that correlated with patient survival.

## Supplementary Information

**Additional file 1: Figure 1.** Effects of CXCL9 and IL-8 plasma levels on patient survival. **Figure 2.** Plasma concentrations of IL-1β and IL-6 Inflammatory markers in plasma of PAH and CTEPH patients at diagnosis and 1-year follow up. **Figure 3.** Inflammatory marker concentration of PH patients at diagnosis and at 1-year follow up are not separated by principal component analysis. **Figure 4.** Contribution of inflammatory markers and clinical parameters to multivariate analyses. **Figure 5.** Combination of inflammatory markers and clinical parameters leads to the best separation of survival in PAH patients. **Table 1.** Antibody kits used for ELISA.

## Data Availability

All data is available if required.

## Abbreviations

PH: Pulmonary hypertension
PAH: Pulmonary arterial hypertension
IPAH: Idiopathic PAH
CTEPH: Chronic thromboembolic pulmonary hypertension
CTD-PAH: Connective Tissue Disease PAH
CHD-PAH: Congenital Heart Disease PAH
HC: Healthy controls
RV: Right ventricular
mRAP: Mean right atrial pressure
PVR: Pulmonary vascular resistance
mPAP: Mean pulmonary arterial pressure
NT-pro BNP: N-terminal pro B-type natriuretic peptide
IL: Interleukin
PI3K: Phosphoinositide 3-kinase
TGFβ: Transforming growth factor
BMPR2: Bone morphogenetic protein receptor type II
Th: T-helper
CXCL: Chemokine (C-X3-C) motif ligand
VEGF: Vascular endothelial growth factor
NYHA: New York Heart Association
PBMCs: Peripheral blood mononuclear cells
PCA: Principal component analysis
MFA: Multiple factor analysis
