# Supplementary material for: Plasma markers in pulmonary hypertension subgroups correlate with patient survival
Source: Respir Res. 2021 May 4;22:137. doi: 10.1186/s12931-021-01716-w (PMC8097895; doi:10.1186/s12931-021-01716-w)
Supplement: Supplementary file 1 — Additional file 1: Figure 1. Effects of CXCL9 and IL-8 plasma levels on patient survival. Figure 2. Plasma concentrations of IL-1β and IL-6 Inflammatory markers in plasma of PAH and CTEPH patients at diagnosis and 1-year follow up. Figure 3. Inflammatory marker concentration of PH patients at diagnosis and at 1-year follow up are not separated by principal component analysis. Figure 4. Contribution of inflammatory markers and clinical parameters to multivariate analyses. Figure 5. Combination of inflammatory markers and clinical parameters leads to the best separation of survival in PAH patients. Table 1. Antibody kits used for ELISA. [file 12931_2021_1716_MOESM1_ESM.docx]

**Additional files.**

**Additional figures**


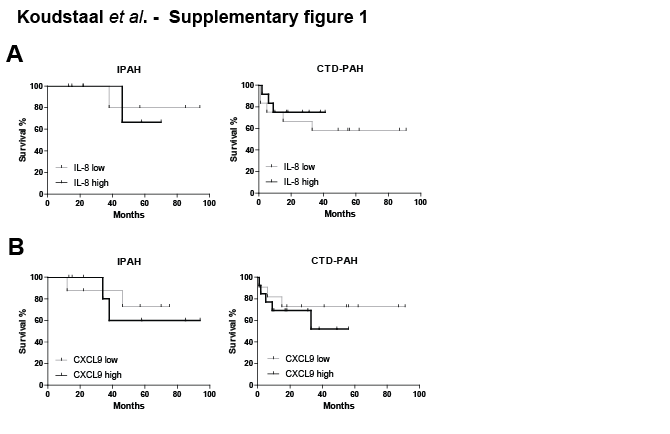


**Additional Figure 1. Effects of CXCL9 and IL-8 plasma levels on patient survival.**

Kaplan-Meyer survival analyses for (A) IL-8-high/low subgroups and (B) CXCL9-high/low of the indicated WHO PH patient subgroups. Statistical analysis was performed using a log-rank (Mantel-Cox) test and a Gehan-Breslow-Wilcoxon test. P values are shown.


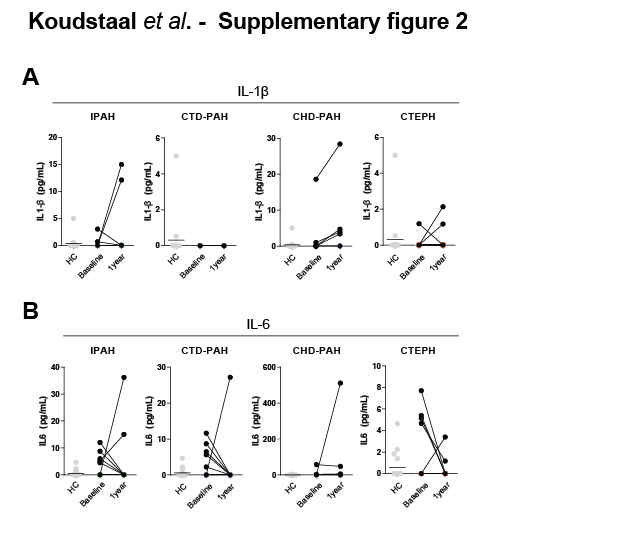


**Additional Figure 2. Plasma concentrations of IL-1β and IL-6 Inflammatory markers in plasma of PAH and CTEPH patients at diagnosis and 1-year follow up.**

(A-B) Paired plasma cytokine measurements by ELISA for interleukin IL-1β (A) and IL-6 (B) at diagnosis and at 1-year follow up for a subset of patients from the indicated WHO patient subgroups, compared with HCs. Data are shown as symbols for individual patients and HCs. Statistical analysis was performed using a Wilcoxon signed-rank test. No significant differences were found.


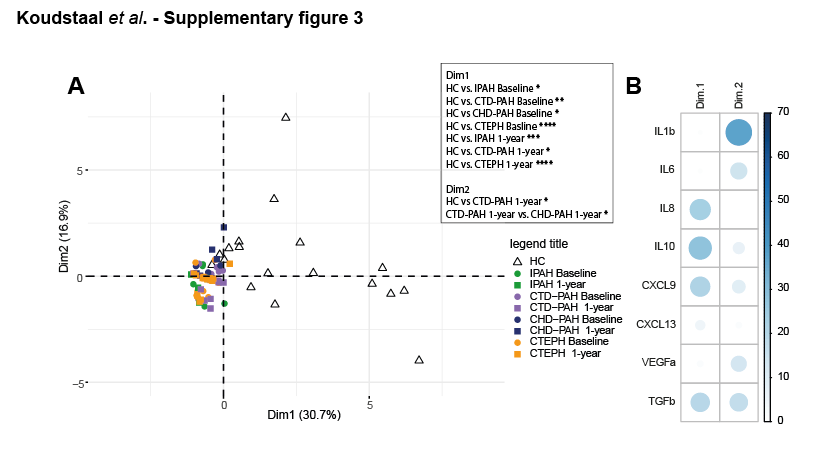


**Additional figure 3. Inflammatory marker concentration of PH patients at diagnosis and at 1-year follow up are not separated by principal component analysis.**

(A) Unsupervised principal component analysis (PCA) of inflammatory markers, measured by ELISA, in plasma of healthy controls and the indicated WHO PH patient subgroups at diagnosis and at 1-year follow-up. PCAs were on log10-transformed and scaled concentrations values; each symbol point represents an individual patient or HC sample. (B) Representation of the contribution in percentages of the variables on the first (Dim.1) and second (Dim.2) principal component of inflammatory markers. The blue color range indicates the contribution to the principal components. Statistical analysis was performed by a one- way ANOVA (Kruskal-Wallis test) combined with a Dunn’s multiple comparison test. * = p <0.05, ** = p<0.01.


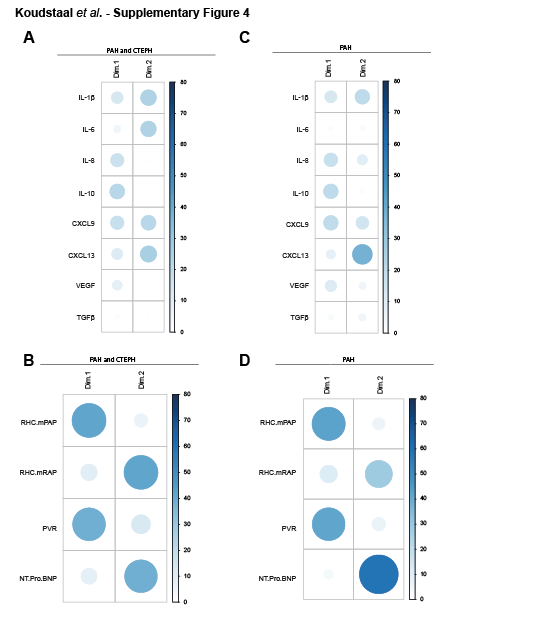


**Additional figure 4.** **Contribution of inflammatory markers and clinical parameters to multivariate analyses.**

(A-D) Representation of the contribution in percentages of the variables on the first (Dim.1) and second (Dim.2) principal component of baseline inflammatory markers of PAH and CTEPH patients (A), clinical parameters of PAH and CTEPH (B), baseline inflammatory markers of solely PAH patients (C) or clinical parameters of solely PAH patients to unsupervised principle component analyses. The blue color range indicates the contribution to the principal components.


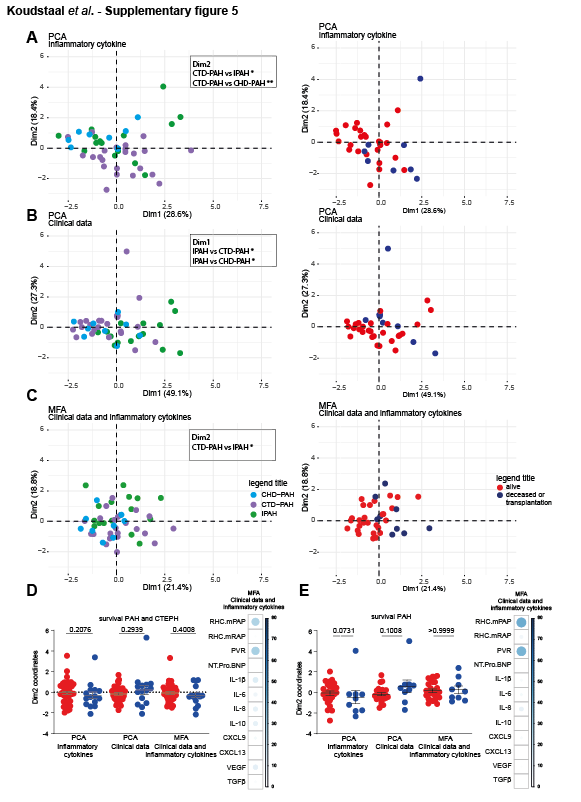


**Additional figure 5. Combination of inflammatory markers and clinical parameters leads to the best separation of survival in PAH patients.**

(A,B) Clustering in PAH patients by unsupervised principal component analysis (PCA) of inflammatory markers measured by ELISA in plasma (A) and of clinical parameters (B), showing the indicated PAH patient subgroups (*left*) or subgroups of survival of >3 years (alive) or <3 years (deceased/transplantation) (*right*). (C) Multiple factor analysis (MFA) combining clinical data and log10 transformed and scaled plasma inflammatory marker concentrations of PAH patients, showing the indicated patient subgroups (*left*) or survival of >3 years (alive) or <3 years (deceased/transplantation) (*right*). (D,E) Dim2 coordinate values of patients with survival of >3 years (alive) or <3 years (deceased/transplantation) for the indicated PCAs and MFAs of PAH and CTEPH patients (D) or PAH patients alone (E) and contribution of the variables for Dim2 to the MFA. Statistical analysis between PAH groups was performed by a one-way ANOVA (Kruskal-Wallis test) combined with a Dunn’s multiple comparison test. * = p <0.05, ** = p<0.01, *** = p<0.001. Separation between survival groups was evaluated using a Mann-Whitney U test on principal component 1 coordinates of alive versus deceased/transplantation. P values are indicated.

**Additional Table 1. Antibody kits used for ELISA.**

| **Cytokine/Chemokine** | **ELISA-kit (Company)** |
| --- | --- |
| IL1- β | R&D duoset ELISA kit human IL-1β (Catalog Numbers: DY201) |
| IL-6 | R&D duoset ELISA kit human IL-6 (Catalog Numbers: DY206) |
| IL-8 | R&D duoset ELISA kit human IL-8 (Catalog Numbers: DY208) |
| IL-10 | R&D duoset ELISA kit human IL-10 (Catalog Numbers: DY217B) |
| CXCL9 | R&D duoset ELISA kit human CXCL9 (Catalog Numbers: DY392) |
| CXCL13 | R&D duoset ELISA kit human CXCL13 (Catalog Numbers: DY801) |
| VEGFa | R&D duoset ELISA kit human VEGFa (Catalog Numbers: DY293B) |
| TGFβ | R&D duoset ELISA kit human TGFβ (Catalog Numbers: DY240) |
